# Motor Matters: Tackling Heterogeneity of Parkinson’s Disease in Functional MRI Studies

**DOI:** 10.1371/journal.pone.0056133

**Published:** 2013-02-13

**Authors:** Štefan Holiga, Karsten Mueller, Harald E. Möller, Tomáš Sieger, Matthias L. Schroeter, Josef Vymazal, Evžen Růžička, Robert Jech

**Affiliations:** 1 Max Planck Institute for Human Cognitive and Brain Sciences, Leipzig, Germany; 2 Department of Neurology and Center of Clinical Neuroscience, Charles University in Prague, Prague, Czech Republic; 3 Department of Cybernetics, Faculty of Electrical Engineering, Czech Technical University in Prague, Prague, Czech Republic; 4 Clinic for Cognitive Neurology & Leipzig Research Center for Civilization Diseases, University of Leipzig and FTLD Consortium, Leipzig, Germany; 5 Department of Radiology, Na Homolce Hospital, Prague, Czech Republic; University Medical Center Groningen UMCG, The Netherlands

## Abstract

To tackle the heterogeneity of Parkinson’s disease symptoms, most functional imaging studies tend to select a uniform group of subjects. We hypothesize that more profound considerations are needed to account for intra/inter-subject clinical variability and possibly for differing pathophysiological processes. Twelve patients were investigated using functional magnetic resonance imaging during visually-guided finger tapping. To account for disease heterogeneity, the motor score and individual symptom scores from the Unified Parkinson’s Disease Rating Scale (UPDRS-III) were utilized in the group-level model using two approaches either as the explanatory variable or as the effect of interest. Employment of the UPDRS-III score and symptom scores was systematically tested on the resulting group response to the levodopa challenge, which further accentuated the diversity of the diseased state of participants. Statistics revealed a bilateral group response to levodopa in the basal ganglia. Interestingly, systematic incorporation of individual motor aspects of the disease in the modelling amended the resulting activity patterns conspicuously, evidencing a manifold amount of explained variability by the particular score. In conclusion, the severity of clinical symptoms expressed in the UPDRS-III scores should be considered in the analysis to attain unbiased statistics, draw reliable conclusions and allow for comparisons between research groups studying Parkinson’s disease using functional magnetic resonance imaging.

## Introduction

Motor symptoms are variably expressed in patients with Parkinson’s disease (PD), i.e. patients with identical summary motor scale scores may express large heterogeneity in specific motor subscores. In fact, various subtypes of the disease have been recognized, such as tremor-predominant PD, akinetic-rigid type PD and PD dominated by postural instability and gait disorder (PIGD), differing in numerous characteristics including the treatment response and rate of progression [1]. The clinical heterogeneity may thus reflect a different proportion of involvement in relevant brain systems [2]. Consequently, the results of functional imaging studies may be obscured by combinations of various involvement types in the examined PD patients’ samples.

The common practice in functional magnetic resonance imaging (fMRI) studies analysing intra- and inter-individual variability in PD patients is to unify the investigated group by involving subjects with a homogenous, narrow distribution of their demographic and clinical characteristics (e.g. age, gender, disease duration, disease laterality, clinical scores). In the current fMRI study, we argue that even within a carefully selected and seemingly uniform population sample, non-apparent between-subject symptomatic variations which require consideration might still be present. We conceived a motor experiment with repeated sessions, including pharmacological intervention, which further increased intra- and inter-subject variability of the patients’ motor behaviour. We hypothesized that systematically accounting for distinctive symptoms of the disease by utilizing clinical scores in random-effect fMRI analyses would have a divergent effect of explaining the variability in the observed data and be apparent in the group-model fit. We considered the variability of PD motor symptoms using the motor part of the Unified Parkinson’s Disease Rating Scale (UPDRS-III) from two different perspectives. In the *UPDRS-III ‘out’* approach, confounding effects of motor symptoms variability are removed to detect unbiased results allowing more straightforward conclusions which are comparable across studies. In the *UPDRS-III ‘in’* approach, the analysis is aimed on revealing potential linear relationships between the particular scores and movement-related brain activity, by detecting the brain regions whose activity correlated with the severity of separate motor symptoms, and further illustrating the general impact of characteristic disease aspects on the fMRI brain responses.

## Methods

### 1. Participants

Twelve male, right-handed patients [mean age 55.9 (SD 6.8) years, range 45–64 years] with idiopathic PD [Hoehn-Yahr stage II-III; duration of disease 12.4 (SD 2.1) years, range 9–15 years] participated in the study. UK PD Society Brain Bank criteria [3] were used for clinical diagnosis. All patients met the criteria for akinetic-rigid type of PD [4]. The patients underwent the experiment in two treatment conditions, first after an overnight withdrawal of dopaminergic medication (OFF condition), and one hour after oral administration of 250 mg of levodopa/25 mg carbidopa (ON condition). The motor score of the Unified Parkinson’s Disease Rating Scale (UPDRS-III) was assessed by a movement disorders specialist (RJ) in each treatment state, immediately preceding the fMRI.

The experiments were performed according to the declaration of Helsinki and all subjects provided a written informed consent prior to their participation. The local ethics committee of the General University Hospital in Prague, Czech Republic approved the protocol of the study.

### 2. Experimental Design

We applied a block-based visuo-motor paradigm of alternating resting and finger-tapping epochs, with each block lasting 10 s. The participants were positioned supine and were instructed to remain motionless with their arms in a resting position while perceiving a red fixation cross and to perform a unilateral single index finger-thumb tap whenever the movement cue (yellow square presented for 100 ms alternating with 1-Hz frequency) appeared on the screen. For each particular medication condition, the session was conducted first with the right hand and was subsequently repeated with the left hand.

In summary, a two-by-two factorial design with the factors ‘hand’ (right, left) and ‘treatment condition’ (off, on) was formed and resulted in four scanning sessions for each participant.

### 3. UPDRS and UPDRS-III Derivatives

The UPDRS is widely used for clinical assessment of impairment and disability in patients with PD [5]. The original scale comprises four distinct parts (Part I: Mentation, Behaviour and Mood; Part II: Activities of Daily Living; Part III: Motor examination; Part IV: Complications) with added Modified Hoehn and Yahr staging and Schwab England scale [6]. The assessor rates each item of the scale with a score (0–4), which is linked to common clinically accepted terms. The sum of the scores indicates the syndrome severity with a higher summary score representing more severe involvement. The UPDRS-III used in this study comprises assessment of PD motor symptoms which expresses the severity of the overall motor impairment.

For each patient in each treatment condition, the total UPDRS-III score and five symptom scores were extracted for further utilization in fMRI modeling. The rigidity score (sum of item 22), akinesia score (sum of items 19, 23–26, 31), and tremor score (sum of items 20–21) were calculated for the right and left side separately, excluding the head and neck-related subitems. The midline score was obtained by summing items 18 and 27–30. The hemibody score was derived as a sum of all UPDRS-III items specific to either left or right extremities (sum of items 20–26) for each hemibody separately. Based on UPDRS III scores, six patients were predominantly affected in their left hemibody, five patients had a predominant right hemibody involvement and one patient showed symmetrical involvement. It should be noted that in one case, rigidity prevailed on the right side despite a higher left hemibody score. In three patients with clear asymmetry in hemibody scores, rigidity was expressed equally on both sides (see Table S1 for more details).

### 4. MRI Acquisitions and Analyses

The MRI data was acquired using a 1.5 T Symphony scanner (Siemens, Erlangen, Germany). A T_2_*-weighted gradient echo echo-planar imaging sequence (*TR*/*TE* = 1000/54 ms) was used for the fMRI. The functional volume consisted of 10 coronal slices, centred around the central sulcus, with 3-mm thick slices, 1-mm slice separation, and a 3×3-mm^2^ nominal in-plane resolution, covering the primary sensorimotor cortex and the basal ganglia. For registration purposes, a *T_1_*-weighted MPRAGE gradient echo acquisition (*TR*/*TI*/*TE*/*FA* = 2140 ms/1100 ms/3.93 ms/15°) was also collected.

A standard fMRI analysis pipeline using SPM8 (Wellcome Trust Centre for Neuroimaging, UCL, London, UK) with Matlab® (R2010b, The MathWorks Inc., Natick, MA, USA) was carried out including realignment, normalization of data using the unified segmentation approach [7] and spatial and temporal filtering [8], [9]. First-level maps were generated using a standard general linear model fit [10] of the pre-processed data with a task-specific predictor. The first-level predictors used statistics which were personalized for every patient, by taking into account their individual movement performance during the task. MR compatible sensory gloves (5^th^ Dimension Technologies, Irvine, CA, USA) were employed to measure the patients’ finger movements and then the recordings were utilized to construct a tailored predictor which was more sensitive to movement deviations and better reflecting movement-related brain activations than conventional hemodynamic response function modelling [11].

**Figure 1:**
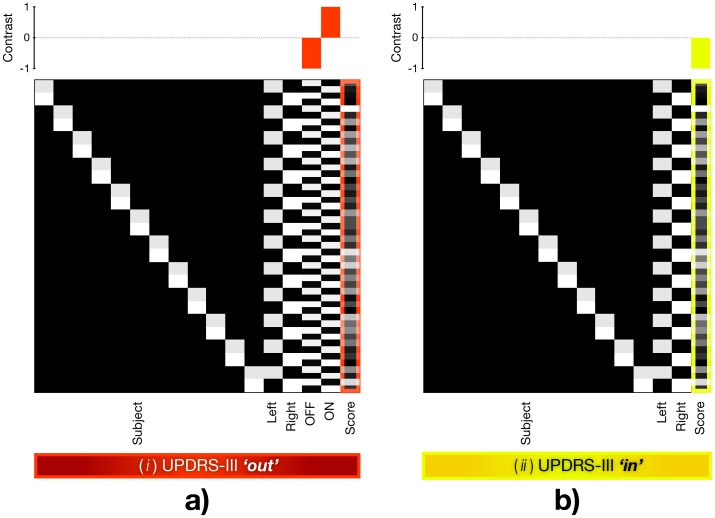
Design matrix and applied contrasts illustrating the idea behind both approaches. a) UPDRS-III *‘out’*: symptom score is utilized as a nuisance factor and the effect of interest represents condition on-off difference. b) UPDRS-III *‘in’*: symptom score is employed as an effect of interest, hence this statistical design reveals correlations between the functional brain responses and the particular motor score (note the contrast above the design matrix). Subject: subject factor. Left: left hand movement. Right: right hand movement. Data obtained in right and left movement tasks was pooled together in each model. OFF: off levodopa condition. ON: on levodopa condition. Score: the UPDRS-III score or symptom score.

To evaluate the group effect of the treatment condition we constructed and fitted the flexible-factorial model. First-level data from the left and right hand sessions were pooled in the model by specifying the ‘hand’ as a factor to increase the statistical power and to profit from the availability of scores specific to left and right body parts (Figure 1). Left symptom scores were used to model data from left hand session and vice versa. In models assessing the UPDRS-III and midline involvement not specific to body parts, the same score was used twice – once for the left and once for the right body part. Two approaches were used to utilize the UPDRS-III scores in the model: (*i*) The *UPDRS-III ‘out’* approach used an additional factor ‘treatment condition’ and the particular aspect of UPDRS-III scores as a covariate forming an explanatory variable (Figure 1a). (*ii*) The *UPDRS-III ‘in’* employed a particular UPDRS-III score or symptom score for correlation with the functional brain responses (Figure 1b). Thus, the *UPDRS-III ‘out’* integrated an experimental factor (2 levels: ON and OFF conditions) in contrast to the *UPDRS-III ‘in’* approach. Accordingly, the employed contrasts differed between the two approaches. While the *UPDRS-III ‘out’* used the ON-OFF contrast as the effect of interest considering the score as the nuisance vector, the *UPDRS-III ‘in’* used the score as the effect of interest (Figure 1). Both *UPDRS-III ‘out’* and *UPDRS-III ‘in’* fitted the identical first-level data. The outcome of personalizing the group-level model using both approaches was systematically observed on the resulting group activity maps, using each particular score separately. To consider the possibility of using multiple scores in one model, Pearson correlation coefficient was calculated for each set of UPDRS-III and symptom scores. The coefficients revealed significant correlations between all scores at the rate of *p*<0.01, except the insignificant correlation between midline and tremor. Correlation between akinesia and tremor was weaker, however still significant (*p*<0.05).

Finally, six *UPDRS-III ‘out’* and six *UPDRS-III ‘in’* models were estimated, employing total UPDRS-III, midline, hemibody, akinesia, rigidity and tremor scores. Furthermore, a conventional model excluding any score was evaluated. The parametric maps resulting from each analysis were corrected for multiple comparisons using the cluster-level family wise error (FWE) correction. Additionally, corrected alpha values (*p*_FWE-corr_), maximum *t*-statistic (*t*_peak_) and number of activated voxels (*k*_E_) were extracted for all significant clusters.

## Results

**Figure 2:**
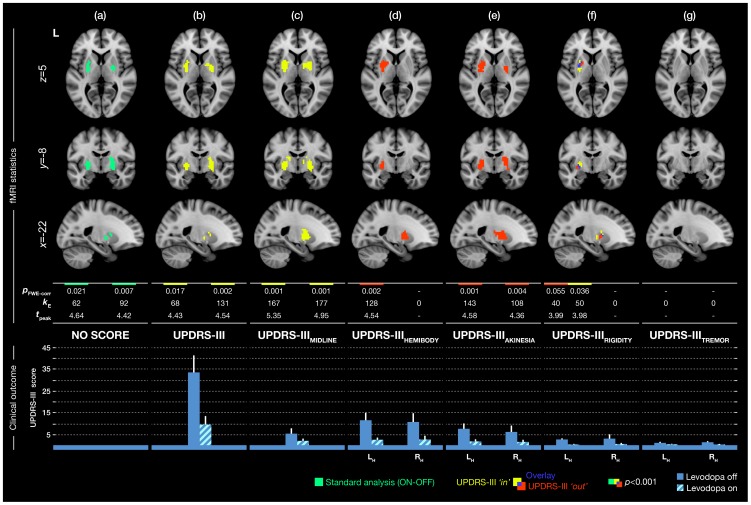
The group response of Parkinson’s disease patients to levodopa, accounting for individual motor aspects of the disease. First column (a) shows results obtained by a conventional analysis not comprising the UPDRS-III scores. Remaining columns represent results obtained by considering a particular score using UPDRS-III as an explanatory/nuisance variable (*UPDRS-III ‘out’*, *red label*) and as the effect of interest to correlate it with the brain responses (*UPDRS-III ‘in’*, *yellow label*). The alpha level was set to *p*<0.001, uncorrected with the cluster extension of *k*≥30 voxels to correct for multiple tests on the cluster level at the rate of *p*_FWE_<0.05. The table displays values obtained by statistical analyses: *p*_FWE-corr_: corrected *p*-value of cluster. *k*_E_: number of activated voxels in cluster. *t*_peak_: peak *t*-statistic value in cluster. A value in the table is assigned to a particular cluster in correspondence with its location in the picture (left/right basal ganglia cluster). Bottom bar-plot depicts average of UPDRS-III scores and subscores used in the analyses with standard deviations. L_H_: left hemibody UPDRS-III score. R_H_: right hemibody UPDRS-III score.

Parametric maps revealed the group responses of PD patients to levodopa treatment in the basal ganglia (Figure 1a). More interestingly, enriching the random-effects model with the specific aspect of UPDRS-III using the *UPDRS-III ‘in’* and *UPDRS-III ‘out’* approaches varied the degree of activity substantially (Figure 2).

The total UPDRS-III score significantly correlated with the functional responses in the basal ganglia regardless of the treatment condition (*‘in’ approach, yellow clusters* on Figure 2b), however completely suppressed the activity corresponding to treatment contrast (ON-OFF), when used as a nuisance variable (*‘out’ approach, no red clusters* on Figure 2b). The midline score resulted in a similar pattern as the total UPDRS-III score in both approaches (Figure 2c). The hemibody score provided no significant correlation (*‘in’ approach, no yellow clusters* on Figure 2d), but equalized hemibody asymmetry of clinical symptoms and revealed a significant difference between treatment conditions (ON-OFF) in the left basal ganglia (*‘out’ approach, red cluster* on Figure 2d). The akinesia score did not provide any significant correlation (*‘in’ approach*, *no yellow clusters* on Figure 2e); however ‘normalized’ the group symptomatically eliciting the most sensitive functional response to levodopa in the basal ganglia (*‘out’ approach, red clusters* on Figure 2e). In particular, the model fit adopting akinesia, despite an additional column in the design matrix, evidenced a 63% increase in the volume of activated clusters (Figure 2e; *k*_E_), and their significance levels (Figure 2e; *p*_FWE-corr_) compared to the conventional analysis (Figure 2a) with no covariate. The rigidity score revealed significant activation in the left basal ganglia with an overlay in both the *UPDRS-III ‘in’* and *UPDRS-III ‘out’* approaches (Figure 1f). Contrarily, the tremor score did not show any significant results at the given threshold for both approaches (Figure 1g).

In summary, the *UPDRS-III ‘in’* approach uncovered significant correlations of functional brain responses in the basal ganglia with the total UPDRS-III score and the midline score (Figure 2b, c), irrespective to the treatment. The *UPDRS-III ‘out’* approach demonstrated that the highest amount of unexplained variability were related to akinesia and rigidity, which most appropriately equalized the group’s clinical picture, and thus delivered the most sensitive group response to levodopa treatment (Figure 2e, f). Moreover, both approaches may lead to positive results even in the same regions, as observed with rigidity, representing the only overlap between the *UPDRS-III ‘in’* and *UPDRS-III ‘out’* (Figure 2f).

## Discussion

In this paper we have introduced the problem of participants’ symptomatic variability in between-subject studies of PD. We revealed the responses to dopaminergic treatment in the basal ganglia, in accordance with previous fMRI work from Kraft et al. [12], Holiga et al. [11] and a positron emission tomography study by Feigin et al. [13]. Furthermore, we demonstrated the results of two distinct approaches revealing this variability by employing the UPDRS-III scores, which remarkably influenced the activation patterns.

A fair number of functional imaging studies examining motor circuitry of PD [14]–[18] embodied the UPDRS-III in statistical analyses to reveal correlations between the clinical presentation of the disease and the blood-oxygen level dependent (BOLD) signal amplitude (Table 1). Correspondingly and noticeably, the *UPDRS-III ‘in’* approach presented here identified a strong relationship between the total UPDRS-III score, midline subscore and BOLD responses in the basal ganglia regardless of the treatment condition. This finding alone should motivate the inclusion of the scores in future motor fMRI studies. The majority of previous studies did not consider the heterogeneity of PD symptoms in the analyses at all (Table 1) [11], [12], [19]–[35]. In addition to examining correlations, we also took advantage of the UPDRS-III scores by means of weeding out variability in the measured data originating from symptomatic deviations within/between subjects (*UPDRS-III ‘out’*). This way we were able to equalize particular clinical symptoms between the investigated patients intra/inter-individually, thus ‘simulated homogeneity’ with respect to a certain symptom. To our knowledge, this and our previous fMRI study [18] are the only studies which considered using UPDRS-III in the analyses to account for the symptomatic variability of PD. Here, akinesia and rigidity were demonstrated as primarily responsive to levodopa treatment, as documented by the scores (Figure 2, bottom bar-plot). Thus, incorporating them quantitatively in statistical prediction of the group response achieved the uppermost sensitive activity pattern representing the patients’ response to levodopa.

**Table 1:** The summary of fMRI studies investigating motor deficits in Parkinson’s disease.

Study	N	Design	Task	UPDRS usedin fMRI	UPDRS subtypesin fMRI	Type of use of clinical picture
Sabatini et al. 2000 [19]	6	block	finger tapping/handmovements	no	–	–
Haslinger et al. 2001 [20]	8	event-related	hand movements	no	–	–
Buhmann et al. 2003 [38]	8	block	finger tappping	no	–	correlation (with measured motor performance)
Wu et al. 2005 [21]	15	block	finger tapping	no	–	–
Macri et al. 2006 [22]	8	block	finger tapping	no	–	–
Holden et al. 2006 [23]	6	block	finger tapping/toe wiggling	no	–	–
Wu et al. 2008 [24]	15	block	hand movements/fingertapping	no	–	–
Mallol et al. 2007 [14]	13	block	finger tapping/handrotations	yes	UPDRS-III	correlation
Palmer et al. 2009 [25]	10	block	hand squeezing/productionof force	no	–	–
Palmer et al. 2009 [26]	10	block	hand squeezing/productionof force	no	–	–
Kraft et al. 2009 [12]	12	block	power grip hand movements	no	–	–
Prodoehl et al. 2010 [15]	20	block	pinch grip	yes	UPDRS-III	correlation
Wu et al. 2010 [16]	15	block	finger movements	yes	UPDRS-III	correlation
Moraschi et al. 2010 [27]	6	block	finger tapping	no	–	–
Tessa et al. 2010 [39]	20	block	hand tapping	no (HY staging used)	–	correlation (with HY), nuisance factor: measured motor performance
Palmer et al. 2010 [28]	10	block	hand squeezing/productionof force	no	–	–
Sen et al. 2010 [29]	5	block	finger tapping	no	–	–
Ng et al. 2010 [30]	10	block	hand squeezing/productionof force	no	–	–
Spraker et al. 2010 [31]	14	block	pinch grip	no	–	–
Kalmar et al. 2011 [32]	10	block	finger movement	no	–	–
Pinto et al. 2011 [33]	9	block	hand movement, speech	no	–	–
Helmich et al. 2011 [40]	38	event-related	motor imagery/saccades production	no	–	correlation (with EMG recordings)
González-García et al. 2011 [34]	17	block	finger tapping	no	–	–
Wu et al. 2011 [17]	18	block	finger movement	yes	UPDRS-III	correlation
Cerasa et al. 2012 [41]	23	block	finger tapping	no (AIMS used)	–	correlation
Martinu et al. 2012 [35]	12	block	button presses	no	–	–
Tessa et al. 2012 [42]	19	block	hand writing	no (HY staging used)	–	correlation (with HY)
Holiga et al. 2012 [11]	12	block	finger tapping	no	–	–
Jech et al. 2012 [18]	12	block	finger tapping	yes	UPDRS-III	nuisance factor: UPDRS-III and oedema; correlation with rigidity and midline score

Search performed in PubMed using keywords “Parkinson’s”, “fMRI”, “motor task”. All studies involving cognitive aspects were excluded. N: Number of studied patients suffering from Parkinson’s disease. UPDRS: Unified Parkinson’s Disease Rating Scale. HY: Hoehn-Yahr score. AIMS: abnormal involuntary movement scale. EMG: Electromyography.

Several interesting findings which weren’t obvious when using the conventional analysis emerged when evaluating UPDRS-III models. As we used the hemibody specific symptom scores in all UPDRS-III models except in the laterally unspecific total UPDRS-III and midline score, we systematically equalized the differential lateral involvement of all symptoms in our sample. Note the particular heterogeneity of lateral involvement of PD in our sample (Table S1). Accounting for the general hemibody score (Figure 2d) delivered a significant difference between the treatment conditions in the left basal ganglia. Therefore we might speculate that some of the symptoms reacting to levodopa were expressed more in the contralateral right side of the body. In case the statistical power is sufficient, this might eventually suggest splitting the study group further according to the lateral dominance of PD symptoms, or explore the results in more detail using more specific subscores.

In the present patient’s group, equalizing the akinesia for each hemibody revealed laterally unspecific and more sensitive levodopa modulation of activity in BG, confirming that our patients were mainly affected by akinesia, and had improvement in both body parts when treated with levodopa (Figure 2e). When assessing the results of both UPDRS-III approaches accounting for rigidity, we observed an overlap between the results of both *UPDRS-III ‘out’* and *UPDRS-III ’in’* approaches in the left basal ganglia (Figure 2f). We may conclude that this area particularly reflects rigidity and simultaneously exhibits sensitivity to levodopa treatment. The asymmetry in basal ganglia activation observed with the ON-OFF medication contrast when considering the hemibody score might be explained by left/right asymmetry in the rigidity score. This is in agreement with the previously observed higher synaptic dopamine increase in the more affected hemisphere when levodopa was administered [36]. Higher activation in the left basal ganglia may then reflect a higher reaction to treatment because of higher expression of rigidity on the right side extremities. Indeed, six patients in our study had rigidity expressed predominantly on the right hemibody, three patients on the left side and for three patients it was manifested symmetrically. All this is particularly interesting, because with a conventional approach alone the sensitivity would be considerably lower and the model would never reveal relationships between various symptoms, laterality or the effects of treatment.

Since PD is considerably heterogeneous, we advocate systematically checking for scores and subscores unquestionably related to the investigated sample using both proposed approaches. This might reveal activity patterns specific to the individual aspects of the disease and potentially lead to unforeseen findings due to increased sensitivity, or suggest further dividing the investigated group of patients in subgroups and analysing them separately. In our case, all patients were categorized as akinetic-rigid, therefore akinesia and rigidity subscore delivered the most sensitive group response. Hence, the proper choice of regressors strongly depends on the population sample studied. It is beneficial to study the outcome of the clinical measures and its variance separately and select the proper *UPDRS-III ‘in’* and/or *UPDRS ‘out’* approach using the particular score or subscore accordingly to research question asked. Moreover, statistical limitations regarding the proper covariate choice must also be considered with this type of analyses. With the exception of one pair, we observed a high degree of correlation between all scores. In a potential multi-score design involving several correlated regressors, besides the reduced degrees of freedom, this would lead to inefficient parameter estimates with high variance, and the incapability to correctly attribute the effect of a particular score to the model fit.

This work is aimed at underlining the strong relationship between the BOLD response and the clinical severity of the disease, but also the importance of considering the intra/inter-subject variability, even in a pre-unified group of PD patients. Because clinical heterogeneity is not clearly defined and is still a matter of debate [37], we advocate using the proposed approaches as leverage for prospective studies involving any group of PD participants for personalizing the statistical evaluations considering the various clinometric involvement. Depending on the research question asked, suitable aspects of UPDRS-III scores can be selected and incorporated in analyses when using *UPDRS-III ‘in’* or *UPDRS-III ‘out’* approaches, to obtain more reliable statistical inferences allowing for unbiased comparisons of results between studies.

## Supporting Information

Table S1 The demographic and clinical characteristics of patients involved in the study.
(PDF)
