# Supplementary material for: Motor Matters: Tackling Heterogeneity of Parkinson’s Disease in Functional MRI Studies
Source: PLoS One. 2013 Feb 13;8(2):e56133. doi: 10.1371/journal.pone.0056133 (PMC3572025; doi:10.1371/journal.pone.0056133)
Supplement: Table S1 — The demographic and clinical characteristics of patients involved in the study. (PDF) [file pone.0056133.s001.pdf]

Supplementary Table S1.

				UPDRS-III*																			
				Total		Midline		Hemibody				Akinesia				Rigidity				Tremor			
				off	on	off	on	off		on		off		on		off		on		off		on	
ID	Age	G	DD	-	-	-	-	L	R	L	R	L	R	L	R	L	R	L	R	L	R	L	R
1	63	M	15	21	5	4	1	7	7	2	1	4	1	1	0	3	4	1	1	1	2	0	0
2	53	M	11	45	9	8	2	17	12	2	2	13	8	1	0	3	3	0	1	1	1	1	1
3	46	M	15	40	13	9	3	11	12	3	4	8	9	3	4	3	3	0	0	0	0	0	0
4	64	M	14	31	2	4	1	11	13	1	0	9	6	1	0	1	2	0	0	1	5	0	0
5	58	M	11	26	11	4	3	6	9	2	4	4	4	1	2	2	4	0	1	1	1	1	1
6	49	M	9	21	9	3	2	10	5	4	2	8	4	4	2	1	1	0	0	1	0	1	0
7	64	M	14	37	11	5	1	8	17	2	6	6	11	2	5	1	4	0	1	1	2	0	0
8	53	M	12	37	11	3	2	18	11	4	4	11	7	3	3	3	2	0	0	4	2	1	1
9	59	M	9	26	6	4	2	12	5	1	0	8	4	0	0	4	1	1	0	0	0	0	0
10	45	M	14	47	21	9	4	17	13	7	6	10	7	5	3	6	5	1	2	2	1	1	1
11	64	M	13	31	10	5	3	11	10	3	2	6	3	1	0	3	4	1	1	2	3	1	1
12	53	M	12	43	10	8	3	11	17	2	4	7	11	1	2	3	5	0	1	1	1	1	1
-	**55.9 (6.8)**	-	**12.4 (2.0)**	**33.8 (8.7)**	**9.8 (4.5)**	**5.5 (2.2)**	**2.3 (0.9)**	**11.6 (3.8)**	**10.9 (3.8)**	**2.8 (1.6)**	**2.9 (2.0)**	**7.8 (2.6)**	**6.3 (3.0)**	**1.9 (1.4)**	**1.8 (1.7)**	**2.8 (1.4)**	**3.2 (1.3)**	**0.3 (0.5)**	**0.7 (0.6)**	**1.3 (1.0)**	**1.5 (1.4)**	**0.6 (0.5)**	**0.5 (0.5)**

Supplementary Table S1. The demographic and clinical characteristics of patients involved in the study. UPDRS-III* - Motor part of Unified Parkinson's Disease Rating Scale. G: gender. DD: duration of the disease (years). OFF: without levodopa medication. ON: with levodopa medication. L: left hemibody. R: right hemibody. The last row displays the average and standard deviation as: mean (SD).
